# Impact of computed tomography (CT) reconstruction kernels on radiotherapy dose calculation

**DOI:** 10.1002/acm2.12994

**Published:** 2020-09-05

**Authors:** Irina Vergalasova, Michael McKenna, Ning Jeff Yue, Meral Reyhan

**Affiliations:** ^1^ Department of Radiation Oncology Rutgers Cancer Institute of New Jersey Rutgers University New Brunswick NJ USA

**Keywords:** CT imaging, dose calculation algorithms, Hounsfield Units, reconstruction kernels

## Abstract

**Purpose:**

To quantitatively evaluate the effect of computed tomography (CT) reconstruction kernels on various dose calculation algorithms with heterogeneity correction.

**Methods:**

The gammex electron density (ED) Phantom was scanned with the Siemens PET/CT Biograph20 mCT and reconstructed with twelve different kernel options. Hounsfield unit (HU) vs electron density (ED) curves were generated to compare absolute differences. Scans were repeated under head and pelvis protocols and reconstructed per H40s (head) and B40s (pelvis) kernels. In addition, raw data from a full‐body patient scan were also reconstructed using the four B kernels. Per reconstruction, photon (3D and VMAT), electron (18 and 20 MeV) and proton (single field) treatment plans were generated using Varian Eclipse dose calculation algorithms. Photon and electron plans were also simulated to pass through cortical bone vs liver plugs of the phantom for kernel comparison. Treatment field monitor units (MU) and isodose volumes were compared across all scenarios.

**Results:**

The twelve kernels resulted in minor differences in HU, except at the extreme ends of the density curve with a maximum absolute difference of 55.2 HU. The head and pelvis scans of the phantom resulted in absolute HU differences of up to 49.1 HU for cortical bone and 45.1 HU for lung 300, which is a relative difference of 4.1% and 6.2%, respectively. MU comparisons across photon and proton calculation algorithms for the patient and phantom scans were within 1–2 MU, with a maximum difference of 5.4 MU found for the 20 MeV electron plan. The 20MeV electron plan also displayed maximum differences in isodose volumes of 20.4 cc for V90%.

**Conclusion:**

Clinically insignificant differences were found among the various kernel generated plans for photon and proton plans calculated on patient and phantom scan data. However, differences in isodose volumes found for higher energy electron plans amongst the kernels may have clinical implications for prescribing dose to an isodose level.

## 1 INTRODUCTION

Computed tomography (CT) imaging is the current backbone of the entire radiotherapy treatment planning process. The scan(s) acquired during simulation set the stage for daily immobilization setup, target volume and organs‐at‐risk delineation, as well as treatment plan dose calculation. Aside from the importance of good image quality for contouring, the electron density information is immensely crucial for accurate radiation modeling of the dose delivered to the patient. A CT calibration curve converts the Hounsfeld unit (HU) values of different materials to electron density (ED) from which treatment planning dose calculation algorithms model the interactions of the incident radiation within the patient in order to calculate the dose. This curve is typically defined during the initial stages of commissioning a treatment planning system.^1^ This curve is specific to the CT scanner from which it is acquired and thus must be regenerated with the installation of any new CT scanners that will be used to image radiotherapy patients.

In the modern‐day era of radiation therapy, the rapid progression of technology has introduced upgraded CT equipment with an abundance of features into the clinic. Some of these new features include metal artifact reduction,^2^, ^3^ extended field‐of‐view,^4^ dual‐energy imaging,^5^, ^6^, ^7^ iterative reconstruction,^8^ and automated tube voltage selection.^9^ The role and impact of these new features has been and continues to be investigated and reported upon in the literature. The more traditionally customizable CT scan parameters such as kilovoltage, current, resolution, slice thickness, field‐of‐view (FOV) and reconstruction algorithm, have been more heavily studied in terms of the induced HU changes and subsequent impact on dose calculation.^10^, ^11^, ^12^, ^13^, ^14^, ^15^, ^16^, ^17^, ^18^, ^19^ These options can be varied through the selection of various anatomic scan protocols pre‐installed onto the scanner. In addition, there is also a large variety of reconstruction kernel options available from the manufacturer to choose from. These kernels impact the resolution and apparent noise of the image, sharpening or smoothing the image depending on the kernel selected. However, currently there exists a lack of guidance on the recommended selection for clinical use, nor is there a thorough quantitative comparison of these different reconstruction kernels and their impact on dose calculation accuracy. It is therefore the purpose of this work to quantify the impact of CT simulation reconstruction kernels on a variety of radiation modalities and dose calculation algorithms with heterogeneity correction.

## 2 MATERIALS AND METHODS

**Fig. 1:**
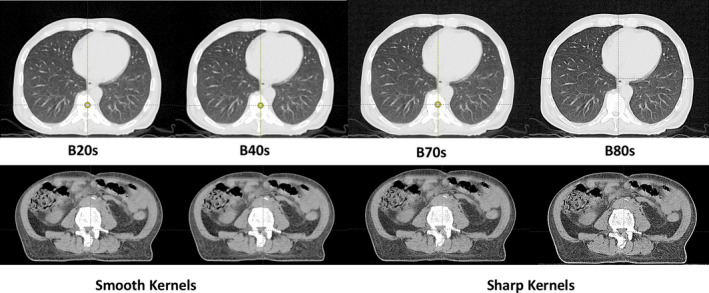
Visual illustration of the selected B (body) reconstruction kernels and their impact on image smoothness/sharpness.

The Gammex electron density (ED) Phantom was initially scanned with the Siemens PET/CT Biograph20 mCT (Siemens Healthineers, Munich, Germany). The acquisition parameters were as follows: 120 kVp, 332 mAs, CareDose4D on, 2 mm slices, 0.8 pitch, and 500 mm field‐of‐view (FOV). The scanner has a drop‐down menu of kernel options to select from: B, H, UH, and D. The B kernels represent body, H kernels represent head, UH kernels represent ultra‐high resolution and D kernels represent a quantitative kernel option, which is meant to provide HU values without the confounding results of additional image processing. Note that not all of these kernel options are available under every scan protocol. The different kernel options selected for HU vs ED study were: B20s, B40s, B60s, B80s, H20s, H40s, H60s, H70s, D20s, D30s, D40s, and D60s. The first digit represents the sharpness of the kernel (lower numbers are smoother and higher numbers are sharper, see Fig. 1 for visual comparison between select B kernels for a sample lung and pelvis scan) and the second digit represents the version of that specific kernel. The letter following the number, f for fast and s for slow, signifies whether a flying focal spot was used during the acquisition (s). The raw data were reconstructed with the twelve listed kernel options in order to generate Hounsfield unit (HU) vs electron density (ED) curves. The numbers following the kernels were chosen to be as close as possible for comparison among the kernels because the exact same ones were not available across B, H and D kernels. A quantitative evaluation was performed to examine the absolute differences in HU amongst these scans. HU was measured directly on the Siemens scanner using a circular ROI. The area of the ROI was approximately 2.64 cm^2^, slightly smaller than the actual plug size. The ROIs do not include the sharp gradient in CT number at the edge of the plug. The same size ROI was used for each plug on each of the different reconstructed scans.

The same Gammex phantom was then scanned with both head and pelvis protocols: 120 kVp, 100 mAs, 2 mm slices, pitch of 0.55/0.8, 500 mm FOV; and reconstructed per H40s (head) and B40s (body) kernels, respectively. These two specific kernels were chosen to be consistent with clinical relevance, as B40 is recommended by the manufacturer’s onsite trainer for clinical use. In addition, a patient dataset was also employed in this study in order to generate results of greater clinical value. Raw data from a full‐body patient scan were reconstructed using the B20s, B40s, B70s, and B80s kernels. B kernels were chosen since H kernels are not anatomically appropriate and D/UH kernels are not always available via drop‐down menu. For each patient scan reconstruction, photon (3D‐CRT and VMAT with 15 MV), electron (en face 18 MeV) and proton (single field) treatment plans were generated using Varian Eclipse dose calculation algorithms Version 11.0.31 (Varian Medical Systems, Palo Alto, California): Anisotropic Analytical Algorithm (AAA_11031), Acuros (AcurosXB_11031), electron Monte Carlo (eMC_11031), and Proton Convolution Superposition (PCS_11031). Note that the proton calibration curve employed for the studied plans was generated using the stoichiometric method, consistent with the current method used clinically. Photon (6 MV) and electron (en face 20 MeV) plans were also simulated to purposely pass through cortical bone vs liver plugs of the phantom for kernel comparison. Treatment field monitor units (MU) and volumes (cm^3^) of specific isodose levels (V105%, V100%, V90%, V50%, V30% and V10%), were compared across all scenarios to assess any differences resulting from reconstruction kernel selection. Volumes (cm^3^) of the selected isodose levels were measured using the “Convert isodose level to structure” feature in ARIA. Note, MU values were not measured for proton plans, as the treatment planning system does not generate MU and instead requires patient‐specific device measurements for MU determination. Plan normalization was selected in the following manner: 3D AP/PA photon plans (AAA) were normalized to isocenter, VMAT plans were normalized as 100% covering 95% of Target Volume, electron plans were normalized to the same point in each plan (similar to traditional electron plan calculations), and proton and Acuros plans were calculated with no plan normalization. All field parameters (and devices) were identical between plan comparisons on different kernel reconstruction scans. For the eMC calculation algorithm, the accuracy was set to 2, which indicates a 2% uncertainty in the D_max_ region. The number of particles per simulation varied from 1,240,000 to 1,300,000 per subfield calculation, with approximately eight subfields per plan.

**Fig. 2:**
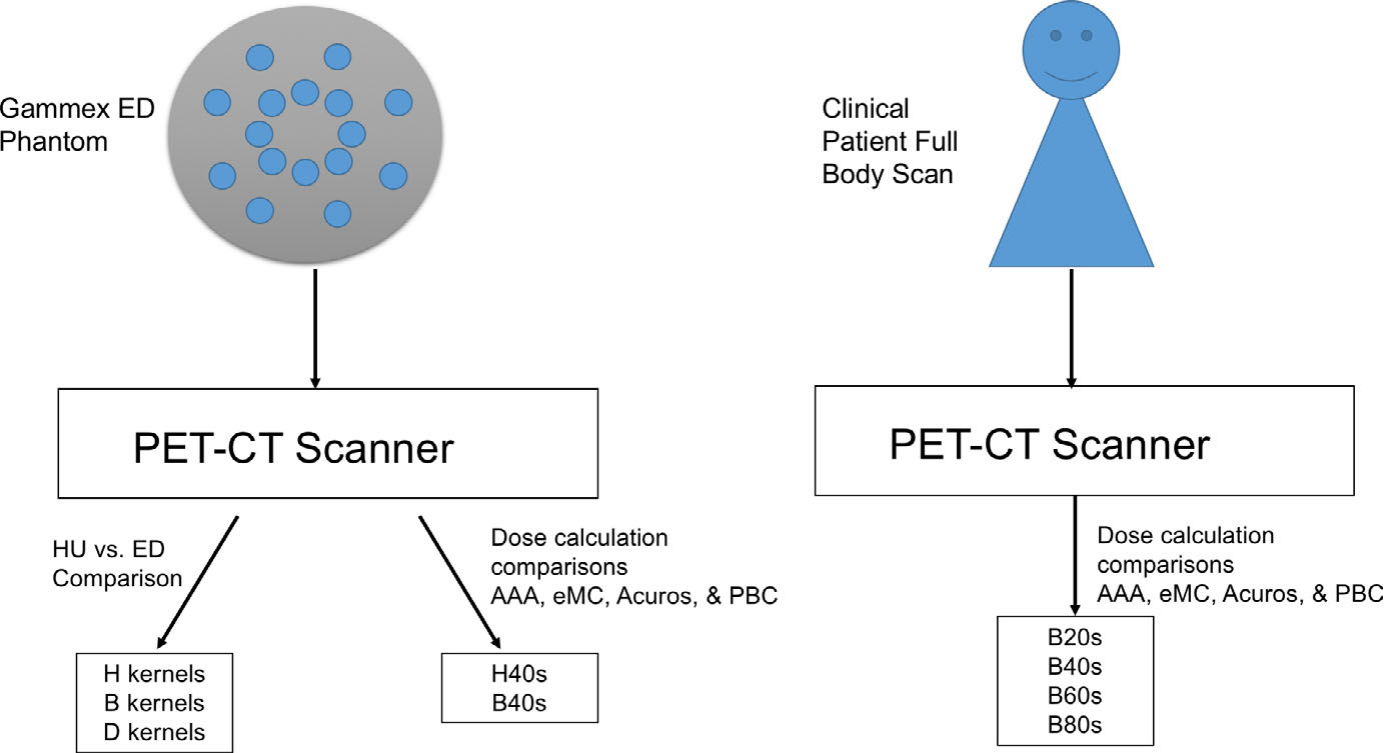
Schematic illustrating the methodology and workflow of the different experiments performed on phantom vs patient datasets, for specific kernel reconstructions and dose calculation algorithm comparisons.

A schematic summarizing the experiment methodology is displayed in Fig. 2.

## 3 RESULTS

**Fig. 3:**
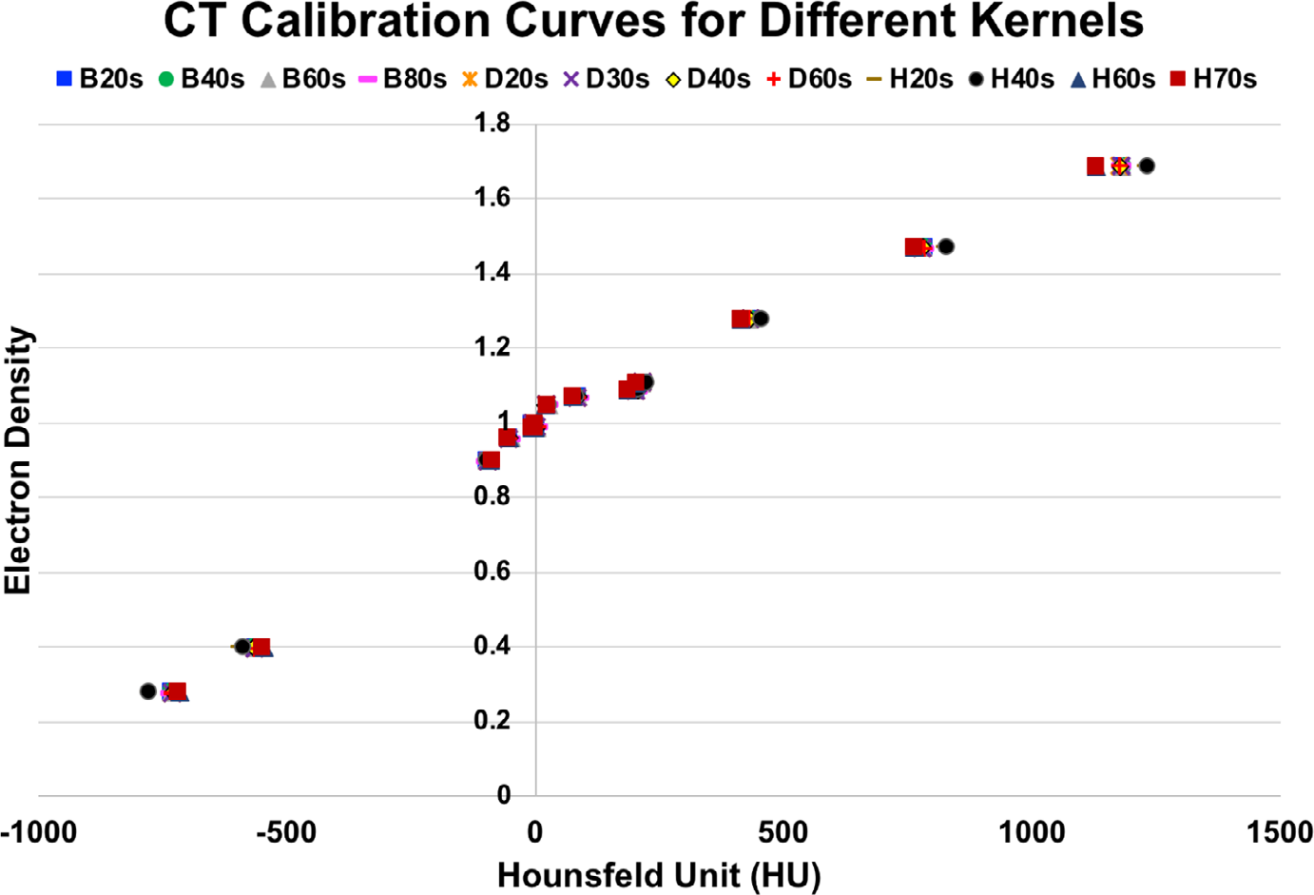
Hounsfeld units (HU) vs electron density (ED) for twelve different reconstruction kernel options (B vs H. vs D kernels) of the Siemens PET/CT Biograph20 mCT.

**Fig. 4:**
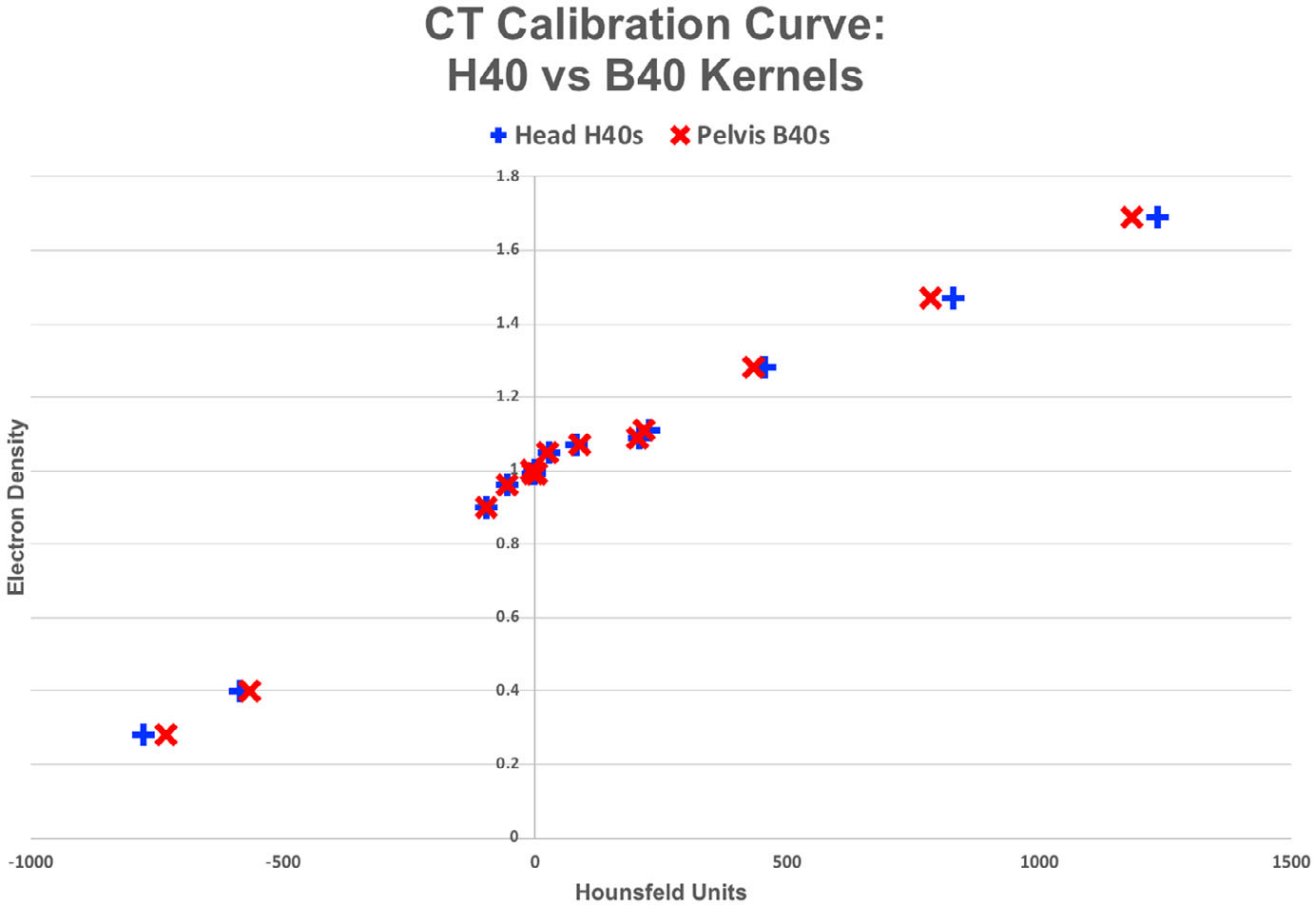
Hounsfeld units (HU) vs electron density (ED) for head and pelvis scans reconstructed with H40s and B40s kernel options of the Siemens PET/CT Biograph20 mCT.

Figure 3 displays HU vs ED calibration curves generated from the twelve different reconstruction kernels. It is evident that the overlapping data points suggest minimal differences were found amongst the kernel options for the extracted HU values for a majority of the standard plug inserts of the Gammex phantom. At the extreme ends of the curve, which correspond to the lowest (air) and highest (bone) density materials, differences on the order of 30‐55 HU can be seen. This is further confirmed with Table 1, which lists the absolute HU values extracted per material for each of the reconstructed scans, with a maximum range between the HUs listed seen to be 55.2 HU. Figure 4 displays a closer look at the calibration curves of the head (H40s) vs pelvis (B40s) vendor‐recommended reconstruction kernels for clinical use, which also shows a difference of between 40 and 50 HU (6%) at the extremes. This is demonstrated quantitatively in Table 2, which lists the absolute HU differences for every material between the two types of generated images. An absolute difference of 49.1HU is seen for cortical bone and 45.1HU for lung 300, which is a relative difference of 4.1% and 6.2%, respectively.

**Table 1:** Measured Hounsfeld Units (HU) of the Gammex phantom plug inserts scanned with the Siemens PET/CT Biograph20 mCT and reconstructed using four B kernels, four D kernels and four H kernels.

B, D, & H kernel reconstructions													
Material	ED	B20s	B40s	B60s	B80s	D20s	D30s	D40s	D60s	H20s	H40s	H60s	H70s
		(HU)	(HU)	(HU)	(HU)	(HU)	(HU)	(HU)	(HU)	(HU)	(HU)	(HU)	(HU)
Lung 300	0.28	−731.2	−731.4	−731	−732	−728.2	−728.7	−726.3	−728.5	−775.9	−776.5	−715	−717.5
Lung 450	0.4	−563.8	−564.9	−563.4	−562.9	−564.2	−564.7	−564.5	−565.2	−592	−584.6	−546	−547.1
Adipose	0.9	−94	−94.7	−95.6	−97.1	−94.3	−94.1	−93.3	−94.5	−94.7	−95.6	−87.6	−86.9
Breast	0.96	−51.3	−53.3	−48.1	−49.9	−51.5	−51.4	−52.3	−51.7	−54.5	−54.5	−51.4	−54.1
Solid water	0.99	1.9	2.0	1.3	1.9	2.0	2.2	2.1	2.1	−0.9	−0.8	−1.6	−1.7
Water	1	−4.4	−5.2	−4.8	−5	−4.4	−5.2	−5.8	−4.1	1.5	1.2	0.7	1.8
Brain	1.05	24.3	25.1	27.3	26.5	24.5	24.1	22.4	26.4	27.2	28.7	23.5	26.1
Liver	1.07	88.2	88.2	87.6	89.6	86	86.8	86.9	86.9	84.2	84.1	75.4	76.4
Inner bone	1.09	206	205.8	202.7	205.7	204.3	204.7	206.8	203.6	207.5	208.1	189	186.4
B−200	1.11	218.1	218.9	215.7	217.4	216.9	218.2	218.9	216.4	225.4	226.1	206.1	205.2
CB2 30%	1.28	435.8	435.2	434.2	438.4	433.3	433	433.3	431.8	454.9	455.9	416.8	415.6
CB250%	1.47	785	785.8	780.8	784	781.1	780.1	781	782.2	828.4	828.7	764.3	764.4
Cort bone	1.69	1184.1	1185.3	1183.3	1182.2	1178.3	1179.2	1179	1177.8	1230.8	1234.4	1129.7	1131.8

**Table 2:** Measured Hounsfeld units (HU) of the Gammex phantom plug inserts scanned with the head and pelvis protocols on the Siemens PET/CT Biograph20 mCT and reconstructed using the H40s and B40s kernels, respectively.

Head (H40s) vs pelvis (B40s) reconstructions				
Material	ED	H40s (HU)	B40s (HU)	Absolute difference
Lung 300	0.28	−776.5	−731.4	45.1
Lung 450	0.4	−584.6	−564.9	19.7
Adipose	0.9	−95.6	−94.7	0.9
Breast	0.96	−54.5	−53.3	1.2
Solid water	0.99	−0.8	2	2.8
Water	1	1.2	−5.2	6.4
Brain	1.05	28.7	25.1	3.6
Liver	1.07	84.1	88.2	4.1
Inner bone	1.09	208.1	205.8	2.3
B‐200	1.11	226.1	218.9	7.2
CB2 30%	1.28	455.9	435.2	20.7
CB250%	1.47	828.7	785.8	42.9
Cort bone	1.69	1234.4	1185.3	49.1

Quantitative MU comparison of photon and electron plans generated on the patient scans reconstructed according to the four different kernel options (B20s, B40s, B70s, B80s) are listed in Table 3. AAA, Acuros and eMC all gave essentially identical MU results across the four different kernel scans, with a maximum difference of 1–2 MU. Creating photon and electron plans with beams passing directly through bone and liver inserts of the Gammex phantom scans reconstructed according to head and pelvis protocols resulted in similarly minor differences for AAA and Acuros. However, for the eMC plans of a 20 MeV beam, Table 4 displays a larger difference of 6MU for the beams passing through cortical bone for H40s vs B40s.

**Table 3:** Calculated monitor units (MUs) per 15 MV photon three‐dimensional (3D)‐CRT (AAA and Acuros), VMAT (AAA) and electron (eMC) plan generated with Varian Eclipse treatment planning system on each of the four reconstructed B kernel scans of the raw patient data.

MU values for full‐body patient scan						
Reconstruction kernel	AAA:15 MV			Acuros:15 MV		eMC
	AP	PA	1‐arc VMAT	AP	PA	18 MeV
B20s	137.6	118.4	399.3	129.0	171.0	269.3
B40s	137.7	118.3	399.3	129.0	171.0	268.8
B70s	137.6	118.2	398.8	129.0	171.0	267.8
B80s	137.6	118.2	398.4	129.0	171.0	267.6

**Table 4:** Calculated monitor units (MUs) per 6 MV photon three‐dimensional (3D)‐CRT(AAA and Acuros) and electron (eMC) plans generated with Varian Eclipse Treatment Planning System per each head (H40s) and pelvis (B40s) reconstructed kernel scan of the Gammex phantom.

MU values for gammex head (H40) & pelvis (B40) protocol scans						
Reconstruction kernel	AAA: 6X		eMC: 20 MeV		Acuros:6X	
	Cortical bone	Liver	Cortical bone	Liver	Cortical bone	Liver
H40s	647.4	600.1	270.6	245.4	623.0	534.9
B40s	645.6	600.6	276.0	249.4	621.9	535.1

Looking at the volumes of specific isodose level distributions (V105%, V100%, V90%, V50%, V30% and V10%), the largest differences (ranging from 15.9 to 26.9 cc absolute differences) are seen in Table 5 for the hotter isodose levels (100% and 105%) amongst the different kernels of the eMC plans on the patient reconstructions. Relative differences of these isodose level volumes range from 5.5% to 44%. However, all of the lower isodose levels (V90%, V50%, V30% and V10%) are within a few ccs of each other across the kernel comparisons. The V105% for AAA also demonstrates an absolute difference 22.6 cc between B20s and B70s, but as a relative difference, is only 3.3%. The rest of the AAA results are within 1–2% of each other. Acuros and PCS plans all show a smaller degree of differences in volume between B20s and B80s scans relative to eMC, with a range of 0.5–8.9 cc. Performing the same experiment with the plans generated on the head vs pelvis scans of the phantom similarly resulted in large volume differences for the 20 MeV beam calculated with eMC (i.e.V90% was 4.28 cc for B40s vs 24.7 cc for H40s), but was not the case for the studied photon and proton plans (Table 6). All of the lower isodose level volumes displayed minor differences amongst all of the treatment modalities.

**Table 5:** Isodose volumes reported in cc for V105%, V100%, V90%, V50%, V30%, and V10% per generated photon (AAA & Acuros), electron (18 MeV) and proton plans generated with Varian Eclipse Treatment Planning System on each of the four reconstructed B kernel scans of the raw patient data.

Dose calculation algorithm	Volume (cc)	Reconstruction kernel			
		B20s	B40s	B70s	B80s
AAA:15X	V105%	690.6	677.6	668	681.7
	V100%	3774.2	3773.2	3774	3787.6
	V90%	5159.7	5157.6	5154.7	5162
	V50%	6334	6353.4	6343.3	6348.4
	V30%	7442.9	7449	7447	7454.3
	V10%	9068.1	9105.6	9071.7	9081.3
Acuros: 15X	V105%	0.01	0.00	0.00	0.02
	V100%	243.9	242.8	244.5	251.8
	V90%	3025.8	3028.9	3026.3	3029.2
	V50%	4334.0	4332.1	4329.1	4331.5
	V30%	5055.1	5068.0	5054.0	5056.8
	V10%	6132.8	6129.0	6130.7	6134.3
eMC: 18 MeV	V105%	60.9	52.2	33.6	34
	V100%	288	284.9	278	272.1
	V90%	431.1	430.1	428.8	427
	V50%	759.4	759.4	760.4	759.4
	V30%	973.7	974.1	975.7	975.7
	V10%	1465.6	1466.3	1469.5	1470.4
PCS: 250 MeV	V105%	6	7.7	5.9	6.7
	V100%	289.9	294.3	289.9	286.9
	V90%	612.2	613.2	612.2	611.7
	V50%	1057.4	1058.9	1056.6	1056.7
	V30%	1239.4	1241.4	1238.3	1238.8
	V10%	1519.5	1521.5	1518	1519.4

**Table 6:** Isodose volumes reported in cc for V105%, V100%, V90%, V50%, V30%, and V10% per generated photon (AAA & Acuros), electron (20MeV), and proton plans generated with Varian Eclipse Treatment Planning System per each head (H40s) and pelvis (B40s) reconstructed kernel scan of the Gammex phantom.

Dose calculation algorithm	Volume (cc)	Cortical bone		Liver	
		H40s	B40s	H40s	B40s
AAA:6X	V105%	176.5	175.8	167.8	167.8
	V100%	187.5	186.7	179.3	179.3
	V90%	212	211.1	202.9	202.9
	V50%	344.7	344.3	346.2	346.6
	V30%	509.8	509.7	517.2	517.1
	V10%	703.7	705.5	689.9	689.7
Acuros: 6X	V105%	394.8	393.8	342.7	343.9
	V100%	424.8	424.2	365.7	366.7
	V90%	490.7	489.5	427.8	430.3
	V50%	873.5	871.6	785.5	786.2
	V30%	1135.8	1135	1100.5	1099.3
	V10%	1390.1	1385.7	1354.2	1353.9
eMC: 20 MeV	V105%	N/A	N/A	N/A	N/A
	V100%	N/A	N/A	N/A	N/A
	V90%	24.7	4.28	1.4	1.6
	V50%	188.9	185.4	177.9	181.0
	V30%	262.0	257.0	268.2	266.9
	V10%	450.2	442.2	447.5	447.9
PCS: 250 MeV	V105%	N/A	N/A	0	0.01
	V100%	71.6	13.33	136.3	139.5
	V90%	284.3	274.5	337.9	333.8
	V50%	1110.4	1106.3	1039	1031.7
	V30%	1179.8	1174.9	1099.3	1091
	V10%	1283.6	1277.2	1188.4	1178.3

**Fig. 5:**
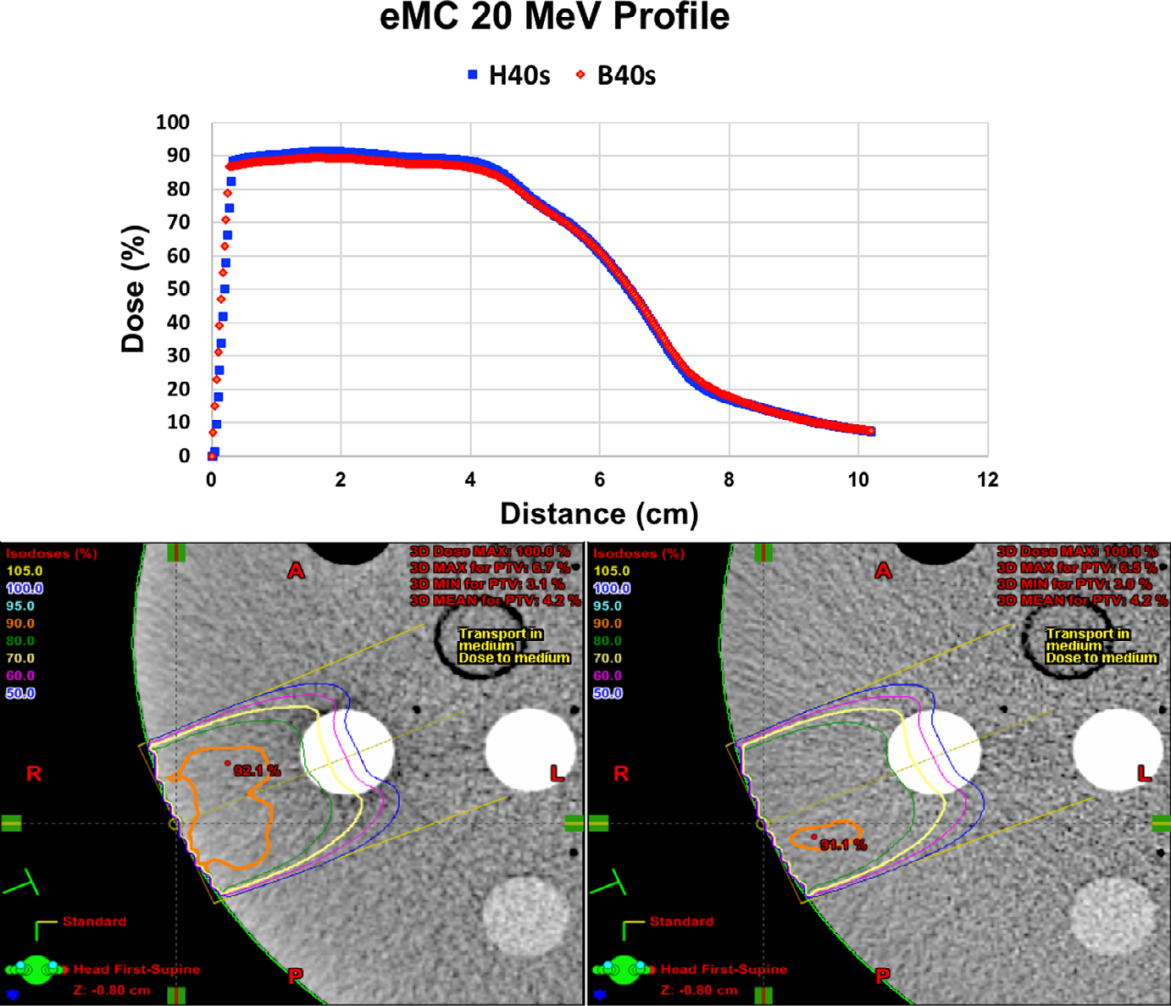
Line profiles (along the yellow dotted line) plotted through the displayed dose distributions of a 20 MeV beam planned with the eMC dose calculation algorithm passing through the cortical bone plug of the Gammex Phantom scanned with the head and pelvis protocols and subsequently reconstructed with the H40s and B40s kernels.

**Fig. 6:**
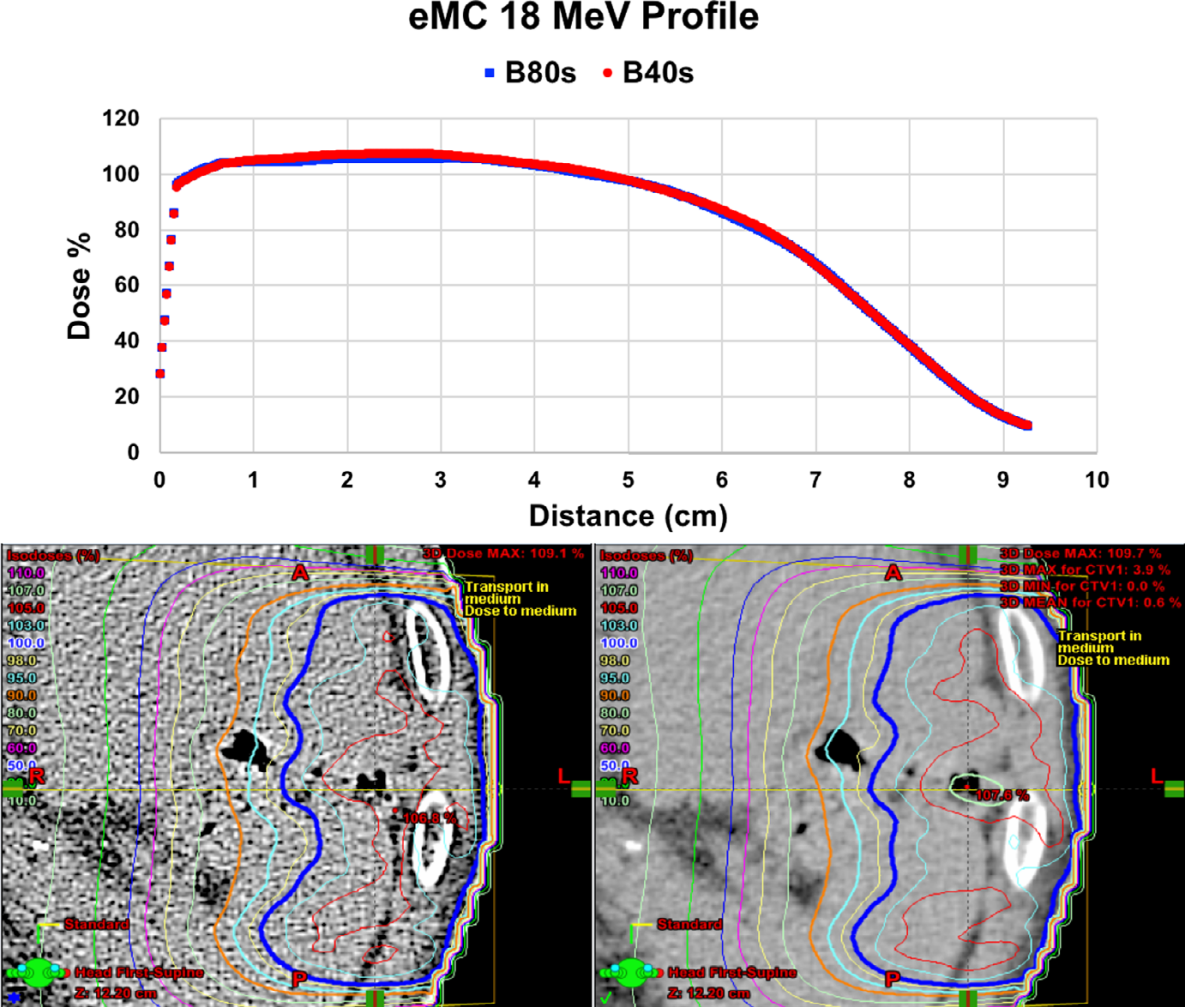
Line profiles (along the yellow dotted line) plotted through the displayed dose distributions of an 18 MeV beam planned with the eMC dose calculation algorithm passing through a patient scan reconstructed with the B80s and B40s kernels.

Based on the results discovered for the higher energy eMC plans listed in Tables 4 and 6, line profiles were plotted for comparison of the 20 MeV plans generated from the head and pelvis kernels in Fig. 5, as well as an axial dose distribution comparison of the two plans. The largest differences between the two plans are evident in the first 4 cm of the line profile, which is further confirmed visually by the different color/shapes of isodose levels in the proximal region to the beam entrance. Line profiles and axial dose distributions are also plotted in Fig. 6 for the 18 MeV eMC beams created on B40s and B80s reconstructions of the patient scan. Although there are apparent differences between the two plans, they are less striking for this energy and scan combination as compared to Fig. 5.

## 4 DISCUSSION

Overall, the results of this study are largely reassuring in that the selection of reconstruction kernel did not have a significant impact on dose calculation algorithms with heterogeneity correction. Comparison of the calibration curves generated from the twelve different reconstruction kernels (B vs D vs H) showed minor differences in HU for the studied plug inserts of the Gammex phantom, with a difference of 40‐55HU at the extreme ends of the density curve (Fig. 3 and Table 1). Further evaluation also demonstrated minimal differences in calculated MU amongst the generated photon, electron and proton plans on these reconstructed patient scans (Table 3). The biggest impact was seen when assessing the higher isodose volumes of V105% and V100%, for the 18 MeV en face electron plans generated on each of the B kernel patient scans. The largest difference found was approximately 27 cc between the B20s and the B80s scans. Similar differences were found when comparing the head and pelvis protocol scans and reconstructions (H40s vs B40s) amongst the 20 MeV en face electron plans: approximately 21 cc difference between V90% and 6 MU difference (i.e. 2.2%) between the plans passing through the cortical bone plug. Although, V105% isodose volumes between B20s and B70s were found to have a 22.6 cc absolute difference for AAA, the relative difference was under 5% and therefore has less clinical consequences because photon plans would not be prescribed to this high of an isodose level. The remainder of the photon and proton plans comparisons between these images did not demonstrate any striking differences in MU or isodose volumes.

Unlike for the AAA photon calculations, the higher energy electron beam differences in the V90% isodose volumes between the kernels may very well have implications for prescribing dose. This has the potential to induce errors for clinical practices that prescribe to a specific isodose volume, such as those used for boosting seromas in the breast after the completion of tangential photon treatment. The degree of error that may be induced for these types of boost plans warrants further investigation.

However aside from the higher energy electrons, the dose calculations were largely similar across the various reconstruction kernels for the different treatment modalities. This may have implications for selecting more optimal image reconstruction kernels per anatomic site allowing easier target or OAR delineation, improving image fusion, and improving the accuracy of automated contouring. In fact, investigating the impact of different reconstruction kernels on the accuracy of automated segmentation algorithms is exactly the goal of future work.

## 5 CONCLUSION

Computed tomography simulation scanner technology has been rapidly evolving and has introduced a wide variety of new features for use in radiation oncology. One such example is the large number of reconstruction kernels available to choose from on the Siemens PET/CT Biograph20 mCT. This study investigated the impact of the available CT reconstruction kernels on dose calculation algorithms with heterogeneity corrections for photons, electrons and protons. A majority of the results demonstrated minor variations in MU calculations and volumes of isodose levels of V10%, V30%, V50%, V90%, V100%, and V105% among the different reconstructed scans. Larger differences in MU and isodose volumes were found for higher energy (18 & 20 MeV) electrons, which may have consequences in the case of prescribing dose to a specific isodose line (i.e. 90%).

## CONFLICT OF INTEREST

The authors have no conflict of interest to disclose.

## References

[1] Fraass B , Doppke K , Hunt M , et al. American Association of Physicists in Medicine Radiation Therapy Committee Task Group 53: quality assurance for clinical radiotherapy treatment planning. Med Phys. 1998;25:1773–1829. 9800687 10.1118/1.598373

[2] Giantsoudi D , De Man B , Verburg J , et al. Metal artifacts in computed tomography for radiation therapy planning: dosimetric effects and impact of metal artifact reduction. Phys Med Biol. 2017;62:R49–R80. 28323641 10.1088/1361-6560/aa5293

[3] Huang JY , Kerns JR , Nute JL , et al. An evaluation of three commercially available metal artifact reduction methods for CT imaging. Phys Med Biol. 2015;60:1047–1067. 25585685 10.1088/0031-9155/60/3/1047 PMC4311882

[4] Cheung JP , Shugard E , Mistry N , Pouliot J , Chen J . Evaluating the impact of extended field‐of‐view CT reconstructions on CT values and dosimetric accuracy for radiation therapy. Med Phys. 2019;46:892–901. 30457170 10.1002/mp.13299

[5] van Elmpt W , Landry G , Das M , Verhaegen F . Dual energy CT in radiotherapy: current applications and future outlook. Radiother Oncol. 2016;119:137–144. 26975241 10.1016/j.radonc.2016.02.026

[6] Goodsitt MM , Christodoulou EG , Larson SC . Accuracies of the synthesized monochromatic CT numbers and effective atomic numbers obtained with a rapid kVp switching dual energy CT scanner. Med Phys. 2011;38:2222–2232. 21626956 10.1118/1.3567509

[7] Landry G , Reniers B , Granton PV , et al. Extracting atomic numbers and electron densities from a dual source dual energy CT scanner: experiments and a simulation model. Radiother Oncol. 2011;100:375–379. 21924780 10.1016/j.radonc.2011.08.029

[8] Dodge CT , Tamm EP , Cody DD , et al. Performance evaluation of iterative reconstruction algorithms for achieving CT radiation dose reduction ‐ a phantom study. J Appl Clin Med Phys. 2016;17:511–531. 27074454 10.1120/jacmp.v17i2.5709 PMC5875046

[9] O'Hora L , Foley SJ . Iterative reconstruction and automatic tube voltage selection reduce clinical CT radiation doses and image noise. Radiography (Lond). 2018;24:28–32. 29306371 10.1016/j.radi.2017.08.010

[10] Davis AT , Palmer AL , Pani S , Nisbet A . Assessment of the variation in CT scanner performance (image quality and Hounsfield units) with scan parameters, for image optimisation in radiotherapy treatment planning. Phys Med. 2018;45:59–64. 29472091 10.1016/j.ejmp.2017.11.036

[11] Ebert MA , Lambert J , Greer PB . CT‐ED conversion on a GE Lightspeed‐RT scanner: influence of scanner settings. Australas Phys Eng Sci Med. 2008;31:154–159. 18697708 10.1007/BF03178591

[12] Guan H , Yin FF , Kim JH . Accuracy of inhomogeneity correction in photon radiotherapy from CT scans with different settings. Phys Med Biol. 2002;47:N223–N231. 12361225 10.1088/0031-9155/47/17/402

[13] Zurl B , Tiefling R , Winkler P , Kindl P , Kapp KS . Hounsfield units variations: impact on CT‐density based conversion tables and their effects on dose distribution. Strahlenther Onkol. 2014;190:88–93. 24201381 10.1007/s00066-013-0464-5

[14] Cozzi L , Fogliata A , Buffa F , Bieri S . Dosimetric impact of computed tomography calibration on a commercial treatment planning system for external radiation therapy. Radiother Oncol. 1998;48:335–338. 9925254 10.1016/s0167-8140(98)00072-3

[15] Beeksma B , Truant D , Holloway L , Arumugam S . An assessment of image distortion and CT number accuracy within a wide‐bore CT extended field of view. Australas Phys Eng Sci Med. 2015;38:255–261. 26048719 10.1007/s13246-015-0353-6

[16] Skrzynski W , Zielińska‐Dąbrowska S , Wachowicz M , Ślusarczyk‐Kacprzyk W , Kukołowicz PF , Bulski W . Computed tomography as a source of electron density information for radiation treatment planning. Strahlenther Onkol. 2010;186:327–333. 20458451 10.1007/s00066-010-2086-5

[17] Li H , Dolly S , Chen H‐C , et al. A comparative study based on image quality and clinical task performance for CT reconstruction algorithms in radiotherapy. J Appl Clin Med Phys. 2016;17:377–390. 10.1120/jacmp.v17i4.5763 PMC5690061 27455472

[18] Chen G‐P , Noid G , Tai A , et al. Improving CT quality with optimized image parameters for radiation treatment planning and delivery guidance. Phys Imaging Radiat Oncol. 2017;4:6–11.

[19] Davis AT , Palmer AL , Nisbet A . Can CT scan protocols used for radiotherapy treatment planning be adjusted to optimize image quality and patient dose? A systematic review. Br J Radiol. 2017;90:20160406. 28452568 10.1259/bjr.20160406 PMC5603945

